# Pyrcca: Regularized Kernel Canonical Correlation Analysis in Python and Its Applications to Neuroimaging

**DOI:** 10.3389/fninf.2016.00049

**Published:** 2016-11-22

**Authors:** Natalia Y. Bilenko, Jack L. Gallant

**Affiliations:** ^1^Helen Wills Neuroscience Institute, University of California, BerkeleyBerkeley, CA, USA; ^2^Department of Psychology, University of California, BerkeleyBerkeley, CA, USA

**Keywords:** canonical correlation analysis, covariance analysis, Python, fMRI, cross-subject alignment, partial least squares regression

## Abstract

In this article we introduce Pyrcca, an open-source Python package for performing canonical correlation analysis (CCA). CCA is a multivariate analysis method for identifying relationships between sets of variables. Pyrcca supports CCA with or without regularization, and with or without linear, polynomial, or Gaussian kernelization. We first use an abstract example to describe Pyrcca functionality. We then demonstrate how Pyrcca can be used to analyze neuroimaging data. Specifically, we use Pyrcca to implement cross-subject comparison in a natural movie functional magnetic resonance imaging (fMRI) experiment by finding a data-driven set of functional response patterns that are similar across individuals. We validate this cross-subject comparison method in Pyrcca by predicting responses to novel natural movies across subjects. Finally, we show how Pyrcca can reveal retinotopic organization in brain responses to natural movies without the need for an explicit model.

## 1. Introduction

Covariance analyses are regarded as one of the simplest approaches for finding similarities across datasets. One type of covariance analysis, known as canonical correlation analysis (CCA), is commonly used in statistics. CCA was first introduced by Hotelling (1936) as a method for finding relationships between two sets of variables. In the subsequent decades it has been extended (Hardoon et al., 2004) and applied in a variety of scientific fields, from climate modeling (Barnett and Preisendorfer, 1987), to computational biology (Yamanishi et al., 2003), to neuroimaging (Hardoon et al., 2007; Correa et al., 2010; Varoquaux et al., 2010). In this article we present Pyrcca (PYthon Regularized Canonical Correlation Analysis), an open-source Python package for performing CCA between two or more datasets. Pyrcca supports CCA with and without regularization and kernelization.

There are several existing software packages that implement CCA. Several implementations are available in MATLAB: Kernel Method Toolbox (https://sourceforge.net/projects/kmbox/), emiCCA (http://www.neuro.uestc.edu.cn/emiCCA.html, Dong et al., 2015), and CCA-fMRI (http://cca-fmri.sourceforge.net/) in SPM (Friston et al., 1994). To our knowledge, there are two implementation of CCA in Python. The cross-decomposition module in scikit-learn (Pedregosa et al., 2011) includes an implementation of CCA. However, it does not include kernelization. Additionally, the package PyKCCA (https://github.com/lorenzoriano/PyKCCA) implements kernel CCA. However, it includes minimal documentation and may not be actively maintained.

In contrast, Pyrcca brings an implementation that supports both kernelization and regularization to the open-source scientific Python ecosystem. Pyrcca includes detailed instructions and examples of general usage and applications to neuroimaging analysis. In this article, we demonstrate application of Pyrcca to neuroimaging data. We analyze publicly available fMRI data recorded from the visual cortex of three subjects who were watching natural movies (Nishimoto et al., 2011, 2014). We then use Pyrcca to find a set of matching brain response patterns across the three subjects. Although this article focuses on use of Pyrcca to analyze neuroimaging data, Pyrcca can be used to analyze any timeseries data. We therefore hope that Pyrcca will also be used in other scientific fields that require timeseries analysis.

This article is structured in the following way. Section 2 introduces mathematical definitions and describes how CCA is computed. Section 3 describes the functionality of the Pyrcca package. Section 4 illustrates the use of Pyrcca with an idealized example: finding linear relationships between two artificially constructed, interdependent datasets. Section 5 illustrates the use of Pyrcca in neuroimaging analysis: performing CCA-based cross-subject comparison on a real fMRI dataset. To facilitate use of Pyrcca we have released the source code on GitHub (http://github.com/gallantlab/pyrcca), along with Jupyter notebooks (Pérez and Granger, 2007) containing code and results presented in Sections 4, 5.

## 2. Canonical correlation analysis

CCA is a method for finding linear correlational relationships between two or more multidimensional datasets. CCA finds a canonical coordinate space that maximizes correlations between projections of the datasets onto that space. CCA shares many mathematical similarities with dimensionality reduction techniques such as principal components analysis (PCA) and with regression methods such as partial least squares regression (PLS).

CCA has many characteristics that make it suitable for analysis of real-world experimental data. First, CCA does not require that the datasets have the same dimensionality. Second, CCA can be used with more than two datasets simultaneously. Third, CCA does not presuppose the directionality of the relationship between datasets. This is in contrast to regression methods that designate an independent and a dependent dataset. Fourth, CCA characterizes relationships between datasets in an interpretable way. This is in contrast to correlational methods that merely quantify similarity between datasets.

CCA has one disadvantage relative to some other methods: it can easily overfit to spurious noise correlations between datasets. However, overfitting can be avoided by curbing the size of the canonical coordinate space, by regularization, or both.

### 2.1. Mathematical definitions

CCA is a method for finding linear relationships between two or more multidimensional datasets. Given two zero-mean datasets **X** and **Y**, $X=(x_{1},x_{2},\ldots x_{n})\in {\mathbb{R}}^{d\times n}$ and $Y=(y_{1},y_{2},\ldots y_{m})\in {\mathbb{R}}^{d\times m}$ (where **x_i_**, **y_i_** are d-dimensional vectors), CCA finds a canonical coordinate space that maximizes correlations between the projections of the datasets onto that space. For each dimension of this coordinate space, there is a pair of projection weight vectors, **a_j_** = (*a*_1*j*_, *a*_2*j*_, …*a*_*nj*_) and **b_j_** = (*b*_1*j*_, *b*_2*j*_, …*b*_*mj*_) called *canonical weights*. The resulting projections of datasets **X** and **Y** onto each dimension of the canonical space are a pair of d-dimensional vectors, **u_j_** = 〈**a_j_**, **X**〉 and **v_j_** = 〈**b_j_**, **Y**〉, that are called *canonical components* or *canonical variates*. CCA maximizes the correlations between each pair of canonical components:

(1) $$\rho_{j}=\max\frac{\langle u_{j},v_{j}\rangle }{\left\|u_{j}\|\|v_{j}\right\|}$$

Theoretically, CCA is solved iteratively by first finding a pair of canonical components **u_1_** and **v_1_**, such that the correlation **ρ_1_** between **u_1_** and **v_1_** is maximized. The second pair of canonical components **u_2_** and **v_2_** is then found, such that the correlation **ρ_2_** between **u_2_** and **v_2_** is maximized, with the constraint that the canonical components **u_2_** and **v_2_** are orthogonal to the preceding canonical components **u_1_** and **v_1_**, respectively. The total number of canonical component pairs is constrained by the dimensionality of datasets **X** and **Y**, and it must be less than or equal to min{*m, n*}. However, to prevent overfitting the number of canonical component pairs that are computed is usually fewer than min{*m, n*}.

In practice, solving CCA iteratively is both computationally intensive and time-consuming. Therefore, it is convenient to to formulate CCA as a generalized eigenvalue problem that can be solved in one shot. To do so, the objective function, which solves for the maximum of the canonical correlation vector, is rewritten in terms of the sample covariance **C_XY_** of datasets **X** and **Y** and the autocovariances **C_XX_** and **C_YY_**:

(2) $$\begin{array}{l} \rho =\max\frac{\langle u,v\rangle }{\left\|u\|\|v\right\|}=\max\frac{\left(a\;\cdot \;X)\;\cdot \;(b\;\cdot \;Y\right)}{\left\|a\;\cdot \;X\|\|b\;\cdot \;Y\right\|}\\ \;=\max\frac{a^{\prime }C_{XY}b}{\sqrt{\left\|a^{\prime }C_{XX}a\|\|b^{\prime }C_{YY}b\right\|}} \end{array}$$

Without constraints on the canonical weights **a** and **b**, the objective function has infinite solutions. However, the size of the canonical weights can be constrained, such that $a^{\prime }C_{xx}a=1$, and $b^{\prime }C_{yy}b=1$. This constraint results in the following Lagrangian:

(3) $$\begin{array}{l} L\left(\lambda ,\text{a},\text{b}\right)={\text{a}}^{\prime }C_{\text{X}\text{Y}}\text{b}-\frac{\lambda_{X}}{2}\left({\text{a}}^{\prime }C_{\text{X}\text{X}}\text{a}-1\right)-\frac{\lambda_{Y}}{2}\left({\text{b}}^{\prime }C_{\text{Y}\text{Y}}\text{b}-1\right) \end{array}$$

The objective function can then be formulated as the following generalized eigenvalue problem:

(4) $$\begin{array}{l} \left(\begin{array}{cc} 0 & C_{\text{X}\text{Y}}\\ C_{\text{Y}\text{X}} & 0 \end{array}\right)\left(\begin{array}{c} \text{a}\\ \text{b} \end{array}\right)=\rho^{2}\left(\begin{array}{cc} C_{\text{X}\text{X}} & 0\\ 0 & C_{\text{Y}\text{Y}} \end{array}\right) \end{array}$$

For CCA with more than two datasets, the generalized eigenvalue problem can be extended simply (Kettenring, 1971):

(5) $$\begin{array}{l} \left(\begin{array}{ccc} 0 & C_{\text{X}\text{Y}} & C_{\text{X}\text{Z}}\\ C_{\text{Y}\text{X}} & 0 & C_{\text{Y}\text{Z}}\\ C_{\text{Z}\text{X}} & C_{\text{Z}\text{Y}} & 0 \end{array}\right)\left(\begin{array}{c} \text{a}\\ \text{b}\\ \text{c} \end{array}\right)=\rho^{2}\left(\begin{array}{ccc} C_{\text{X}\text{X}} & 0 & 0\\ 0 & C_{\text{Y}\text{Y}} & 0\\ 0 & 0 & C_{\text{Z}\text{Z}} \end{array}\right) \end{array}$$

### 2.2. Regularized CCA

If datasets **X** and **Y** have dimension *d* < min {*m, n*} then CCA is ill-posed and the generalized eigenvalue problem cannot be solved without regularization. Imposing L2 regularization resolves this problem by constraining the norms of canonical weights **a** and **b**. Imposing the L2 penalty maintains the convexity of the problem and the generalized eigenvalue formulation. However, regularization relaxes the orthogonality constraint of the canonical components. Regularization is incorporated in the objective function:

(6) $$\begin{array}{l} \rho =\max\frac{{\text{a}}^{\prime }C_{\text{X}\text{Y}}\text{b}}{\sqrt{\left({\text{a}}^{\prime }C_{\text{X}\text{X}}\text{a}+\lambda ||\text{a}|{|}^{2}\right)\cdot \left({\text{b}}^{\prime }C_{\text{Y}\text{Y}}\text{b}+\lambda ||\text{b}|{|}^{2}\right)}} \end{array}$$

The generalized eigenvalue problem is also modified to incorporate regularization:

(7) $$\begin{array}{l} \left(\begin{array}{cc} 0 & C_{\text{X}\text{Y}}\\ C_{\text{Y}\text{X}} & 0 \end{array}\right)\left(\begin{array}{c} \text{a}\\ \text{b} \end{array}\right)=\rho^{2}\left(\begin{array}{cc} C_{\text{X}\text{X}}+\lambda I & 0\\ 0 & C_{\text{Y}\text{Y}}+\lambda I \end{array}\right) \end{array}$$

Regularized CCA is mathematically similar to partial least squares regression (PLS). Compare to the objective function of CCA (Equation 2) the objective function that is optimized in PLS:

(8) $$\begin{array}{l} \rho =\max\frac{{\text{a}}^{\prime }C_{\text{X}\text{Y}}\text{b}}{\sqrt{{\text{a}}^{\prime }\text{a}{\text{b}}^{\prime }\text{b}}} \end{array}$$

Analogously to CCA, PLS can be solved as a generalized eigenvalue problem:

(9) $$\begin{array}{l} \left(\begin{array}{cc} 0 & C_{\text{X}\text{Y}}\\ C_{\text{Y}\text{X}} & 0 \end{array}\right)\left(\begin{array}{c} \text{a}\\ \text{b} \end{array}\right)=\rho^{2}\left(\begin{array}{cc} I & 0\\ 0 & I \end{array}\right) \end{array}$$

The difference between CCA and PLS is that the PLS objective function is not normalized by the autocovariance of the data. Thus, PLS can be thought of as an asymptotically large regularization of CCA, where **C_XX_** + λ*I* and **C_YY_** + λ*I* are dominated by λ*I*.

### 2.3. Kernelized CCA

Sometimes it is useful to project the data onto a high-dimensional space before performing CCA. This is known as the kernel trick. If a linear kernel function such as an inner product is used, then kernelization is a form of dimensionality reduction. If a nonlinear kernel function such as a polynomial or a Gaussian kernel is used, then kernelization allows the analysis to capture nonlinear relationships in the data.

To perform kernel CCA, a kernel function ϕ(**X**) is chosen and the data are projected onto the kernel space:

$$\begin{array}{l} \phi :X=(x_{1},x_{2},\ldots x_{n})\to \phi (X)=(\phi_{1}(X),\phi_{2}(X),\ldots ,\phi_{K}(X)),\\ \text{ where }n<K. \end{array}$$

Kernel projections of the data, **K_X_** and **K_Y_**, are used instead of datasets **X** and **Y** to solve CCA. The canonical components **u** and **v** are projections of **K_X_** and **K_Y_** onto the canonical space. The eigenvalue problem is reformulated in terms of **K_X_** and **K_Y_**:

(10) $$\begin{array}{l} \left(\begin{array}{cc} 0 & K_{\text{X}}K_{\text{Y}}\\ K_{\text{Y}}K_{\text{X}} & 0 \end{array}\right)\left(\begin{array}{c} \text{a}\\ \text{b} \end{array}\right)=\rho^{2}\left(\begin{array}{cc} K_{\text{X}}^{2} & 0\\ 0 & K_{\text{Y}}^{2} \end{array}\right) \end{array}$$

If the kernel function used for kernel CCA is invertible then regularization must be used. This is because a trivial and undesirable solution can be found by setting **a** = 1 and solving for **b**: $b=\frac{1}{\lambda }K_{Y}{}^{-1}K_{X}$ (or vice versa). With regularization this trivial solution is avoided. The objective function for regularized kernel CCA becomes:

(11) $$\begin{array}{l} \rho =\max\frac{{\text{a}}^{\prime }K_{\text{X}}K_{\text{Y}}\text{b}}{\sqrt{\left({\text{a}}^{\prime }K_{\text{X}}^{2}\text{a}+\lambda ||\text{a}|{|}^{2}\right)\cdot \left({\text{b}}^{\prime }K_{\text{Y}}^{2}\text{b}+\lambda ||\text{b}|{|}^{2}\right)}} \end{array}$$

The generalized eigenvalue problem is reformulated to solve regularized kernel CCA:

(12) $$\begin{array}{l} \left(\begin{array}{cc} 0 & K_{\text{X}}K_{\text{Y}}\\ K_{\text{Y}}K_{\text{X}} & 0 \end{array}\right)\left(\begin{array}{c} \text{a}\\ \text{b} \end{array}\right)=\rho^{2}\left(\begin{array}{cc} K_{\text{X}}^{2}+\lambda I & 0\\ 0 & K_{\text{Y}}^{2}+\lambda I \end{array}\right) \end{array}$$

While kernel CCA is advantageous for capturing nonlinear relationships, it presents additional challenges due to selection of the kernel function and regularization coefficient, as well as difficulty in the interpretation of the kernel canonical components.

### 2.4. Cross-dataset prediction with CCA

CCA finds a symmetric set of common dimensions across datasets. These dimensions are the canonical components. Unlike regression methods, CCA does not assume a causal relationship between datasets. Instead, it assumes that the datasets are dependent on one or more common latent variables. However, it is possible to reframe CCA as a predictive model. Once CCA is estimated between two or more datasets, and the canonical components and canonical weights are estimated, new samples from one of the datasets can be predicted from the canonical weights and new samples from the other datasets. This cross-dataset prediction is accomplished by projecting new samples from all but one dataset onto the canonical space. The new samples from the remaining dataset can then be predicted as the dot product of the inverse of the canonical weights for that dataset and the new samples from the other datasets projected onto the canonical space via the canonical weights:

(13) $$\begin{array}{l} {\text{Y}}_{predicted}={\text{b}}^{-1}\cdot \left({\text{a}}^{\prime }{\text{X}}_{novel}\right) \end{array}$$

If the observed novel data for the remaining dataset are available, the accuracy of the cross-dataset prediction can be quantified by correlating the predicted samples with the actual samples along each dimension of the remaining dataset.

(14) $$\begin{array}{l} \text{accuracy}=corr\left({\text{Y}}_{predicted},{\text{Y}}_{novel}\right) \end{array}$$

Cross-dataset prediction relies on inverting the canonical weight matrix. However, in most cases the canonical weight matrix will not be positive definite and therefore it will not be invertible. In this case, a pseudoinverse must be used to invert the canonical weights. For stability, the pseudoinverse can be regularized. In Pyrcca, we provide the option for pseudoinverse regularization using the spectral cutoff method, in which small eigenvalues are discarded during singular value decomposition. Other regularization methods, such as L2 penalty, could also be used, though they are not currently implemented in Pyrcca.

## 3. Pyrcca functionality

Pyrcca is a Python package for performing CCA. It is hosted in a public GitHub repository (http://github.com/gallantlab/pyrcca). For simplicity, the package is defined in one file: rcca.py. Pyrcca requires three third-party libraries: NumPy (Van Der Walt et al., 2011), SciPy (Jones et al., 2001), and h5py (Collette, 2013).

**Figure 1:**
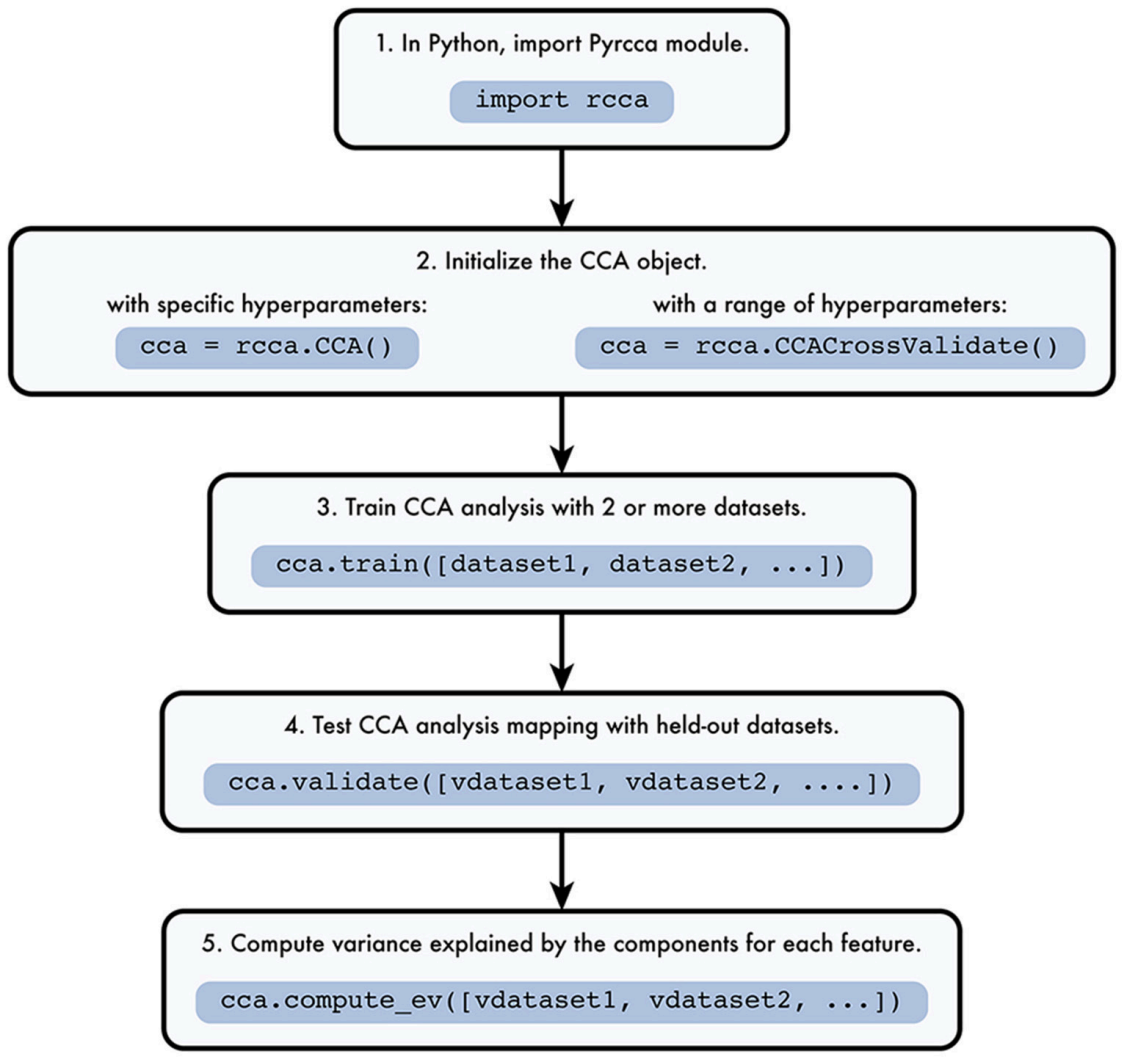
**Pyrcca workflow**. [1] The Pyrcca Python module is imported using the command import rcca. [2] A CCA object is initialized in one of two ways. If specific hyperparameters (the regularization coefficient and the number of canonical components) are used, the rcca.CCA class object is initialized. If the hyperparameters are chosen empirically using cross-validation, then the rcca.CCACrossValidate class object is initialized. [3] The CCA mapping is estimated using the rcca.train() method with training datasets dataset1, dataset2, etc. [4] Once the CCA mapping is estimated, its accuracy can be tested using the method rcca.validate() with held-out datasets vdataset1, vdataset2, etc. [5] The variance explained by each estimated canonical component for each feature in the held-out data is computed using the method rcca.compute_ev() with held-out datasets vdataset1, vdataset2, etc.

The Pyrcca workflow is depicted in Figure 1. The analysis begins by instantiating one of two analysis classes defined in rcca.py, rcca.CCA or rcca.CCACrossValidate. The rcca.CCA class allows the user to predefine two hyperparameters: the regularization coefficient and the number of canonical components. The rcca.CCACrossValidate class allows the user to estimate these two hyperparameters empirically by using grid search with cross-validation.

Both rcca.CCA and rcca.CCACrossValidate classes inherit from the base parent class rcca._CCABase. The class rcca._CCABase is not used for analysis, but defines attributes and methods shared by its two child classes.

### 3.1. Pyrcca instantiation and attributes

The code below shows how the rcca.CCA class is instantiated with the regularization coefficient 0.1, and with 5 canonical components to be computed.


  import rcca
  cca = rcca.CCA(reg=0.1, numCC=5)


If the attributes reg and numCC are not instantiated explicitly, the default values are reg = 0.0 (no regularization) and numCC = 10.

The code below shows how the rcca.CCACrossValidate class is instantiated with three regularization coefficient values: 10^−3^, 10^−2^, 10^−1^, and with three numbers of canonical components to be computed: 2, 3, 4.


  import rcca
  cca = rcca.CCACrossValidate(regs=[1e-3,
       1e-2, 1e-1], numCC=[2, 3, 4])


If the attributes regs and numCCs are not instantiated explicitly, the default values are reg = numpy.array(numpy.logspace(−3, 1, 10)) (ten logarithmically spaced values between 1 × 10^−3^ and 1 × 10^1^) and numCC = numpy.arange(5, 11) (five consecutive integer values between 5 and 10). The ranges of hyperparameter values can be passed to the rcca.CCACrossValidate class object as either lists or NumPy arrays.

Four additional attributes can be specified at instantiation for both classes rcca.CCA and rcca.CCACrossValidate: kernelcca, ktype, cutoff, and verbose.

The Boolean attribute kernelcca specifies whether kernelization should be used (described in Section 2.3). The attribute is set to True by default, which means kernelization is used. If kernelcca is set to True, the string attribute ktype specifies the type of kernel function that is used. There are two accepted values for ktype. The default value is 'linear', which specifies that a linear kernel function (i.e., the inner product of the data) is used. The other accepted values are 'gaussian' and 'poly'. The value 'gaussian' specifies that a Gaussian kernel function is used. The variance for the Gaussian kernel function is specified using an additional attribute gausigma, set to 1.0 by default. The value 'poly' specifies that a polynomial kernel fucntion is used. The degree of the polynomial kernel function is specified using an additional attribute degree, set to 2 by default.

The floating point attribute cutoff controls evaluation of cross-validation results in Pyrcca. As described in Section 2.4, CCA can be used for cross-dataset prediction across datasets, which requires computing a pseudoinverse of the canonical weight matrix if that matrix is not invertible. The pseudoinverse can be regularized using the spectral cutoff method. The attribute cutoff specifies the eigenvalue threshold used for regularization. Eigenvalues smaller than cutoff are set to zero during singular value decomposition. The default value of cutoff is 0.0 (i.e., no regularization).

The Boolean attribute verbose determines whether status messages about the analysis are returned to the console. The default value is True, which means that the status messages are returned. If verbose is set to False, the status messages are suppressed.

When the rcca.CCACrossValidate class is used, two additional attributes can be specified to control how the grid search with cross-validation is implemented: numCV and select.

The integer attribute numCV specifies the number of cross-validation iterations used for testing each set of hyperparameters (the regularization coefficient and the number of canonical components). The numCV attribute has a default value is 10.

The floating point attribute select determines how the accuracy metric is computed during cross-validation. To evaluate each set of hyperparameters, a CCA mapping is estimated for a subset of the data during each cross-validation iteration, and cross-dataset prediction is performed on the held-out data. The predictions are correlated with the actual held-out data. The prediction performance is quantified by taking the mean of the correlations for a portion of the samples that are predicted most accurately. The attribute select specifies the proportion of the samples that is used. The default value of the select attribute is 0.2, meaning that 0.2 of the samples are used. Using a subset of the samples to compute the accuracy metric is advantageous when a large number of the samples are noisy.

### 3.2. Pyrcca implementation and methods

After a CCA object is created with the attributes defined above, the analysis is run using the train() method. After CCA training is complete, the resulting canonical mapping can be tested using the validate() method, which performs cross-dataset prediction with novel data. An additional evaluation of the canonical mapping can be implemented using the compute_ev() method, which quantifies the variance explained by each canonical component in novel data. The methods save() and load() are used for saving the analysis on disk in the HDF5 format, and for loading a previously saved analysis into memory, respectively. We describe each of these methods in detail below.

#### 3.2.1. Pyrcca training

The train() method estimates the CCA mapping between two or more datasets. The datasets are passed to the method as a list of NumPy two-dimensional arrays (number of samples by number of dimensions). The train() method is the only method that differs in its implementation between the two CCA object classes, rcca.CCA and rcca.CCACrossValidate.

When using the rcca.CCA object class, the analysis is only run once with predetermined hyperparameters (the regularization coefficient and the number of canonical components). The code below shows how training is implemented for two datasets after instantiating the rcca.CCA class object with regularization coefficient 0.1 and 5 canonical components:


  import rcca
  cca = CCA(reg = 0.1, numCC = 5)
  cca.train([dataset1, dataset2])


When using the rcca.CCACrossValidate object class, grid search with Monte Carlo cross-validation is first used to find the optimal set of hyperparameters. During each cross-validation iteration, randomly selected 20% of the training data, comprised of blocks of 10 consecutive samples, is held out. CCA mapping is done using the remaining 80% of the training data, for each possible set of the hyperparameter values. Then, cross-dataset prediction is performed using the estimated CCA mapping and the held-out 20% of the training data (for details on cross-dataset prediction, see Section 2.4).

The accuracy of prediction is quantified for each cross-validation iteration in order to choose the optimal hyperparameters. The mean of the highest correlations between predicted and actual samples is used to quantify the prediction accuracy. The portion of the correlations used in this computation is specified using the select attribute. The pair of hyperparameters with the highest cross-dataset prediction accuracy is then chosen, and CCA is run on all training data with those values.

The code below shows how training is implemented in Pyrcca for three datasets. First, a rcca.CCACrossValidate class object is instantiated with three possible regularization coefficient values: 10^−3^, 10^−2^, and 10^−1^, and with three possible numbers of canonical components: 2, 3, and 4.


  import rcca
  cca = rcca.CCACrossValidate(regs=[1e-3,
       1e-2, 1e-1], numCC=[2, 3, 4])
  cca.train([dataset1, dataset2, dataset3])


The train() method adds three new attributes to the CCA object: comps (canonical components), ws (canonical weights), and cancorrs (canonical correlations). For the rcca.CCACrossValidate object, the train() method also adds attributes best_reg (optimal regularization coefficient) and best_numCC (optimal number of canonical components).

#### 3.2.2. Pyrcca validation

The validate() method assesses the CCA mapping that was estimated using the train() method by performing cross-dataset prediction with test data and canonical weights (for details on cross-dataset prediction, see Section 2.4). The test data are passed to the method as a list of NumPy two-dimensional arrays (number of samples by number of dimensions), in the same order as the training data. This method is the same for the rcca.CCA and rcca.CCACrossValidate object classes. The code below shows how validation is implemented in Pyrcca:


  import rcca
  cca = rcca.CCACrossValidate(regs=[1e-3,
       1e-2, 1e-1], numCC=[2, 3, 4])
  cca.train([dataset1, dataset2, dataset3])
  cca.validate([test_dataset1, test_dataset2,
       test_dataset3])


The validate() method adds two attributes to the CCA object: preds (cross-dataset predictions) and corrs (correlations of the cross-dataset predictions and the actual test data).

#### 3.2.3. Computing explained variance in Pyrcca

The compute_ev() method estimates the variance explained (**R**^2^) in the test data by each of the canonical components. The test data are passed to the method as a list of NumPy two-dimensional arrays (number of samples by number of dimensions), in the same order as the training data. This method is the same for the rcca.CCA and rcca.CCACrossValidate object classes. The code below shows how variance explained is estimated:


  import rcca
  cca = rcca.CCACrossValidate(regs=[1e-3,
       1e-2, 1e-1], numCC=[2, 3, 4])
  cca.train([dataset1, dataset2, dataset3])
  cca.validate([test_dataset1, test_dataset2,
       test_dataset3])
  cca.compute_ev([test_dataset1, test_dataset2,
       test_dataset3])


The compute_ev() method adds the attribute ev (variance explained for each component, for each dimension of the test data).

#### 3.2.4. Saving and loading the analysis in Pyrcca

The save() method saves all the attributes in the Pyrcca object to an HDF5 file. The load() method loads attributes from an HDF5 file with a Pyrcca analysis saved using the save() method. Both the save() and the load() method are the same for the rcca.CCA and rcca.CCACrossValidate object classes. The code below shows how the analysis described above can be saved to disk and then loaded from disk in a new session:


  import rcca
  cca = rcca.CCACrossValidate(regs=[1e-3,
       1e-2, 1e-1], numCC=[2, 3, 4])
  cca.train([dataset1, dataset2, dataset3])
  cca.validate([test_dataset1, test_dataset2,
       test_dataset3])
  cca.compute_ev([test_dataset1, test_dataset2,
       test_dataset3])
  cca.save(“Pyrcca_analysis.hdf5”)
  # New session
  import rcca
  cca = rcca.CCACrossValidate()
  cca.load(“Pyrcca_analysis.hdf5”)


## 4. Pyrcca usage example

To illustrate the use of Pyrcca with realistic data, we constructed two linearly dependent datasets and used Pyrcca to find linear relationships between them. The goal of this analysis was to evaluate whether Pyrcca can identify and characterize the relationship between two artificially constructed datasets. The rows of the datasets correspond to the number of samples in the datasets, and the columns correspond to the number of dataset dimensions. In the specific example of cross-subject comparison of BOLD responses, described in Section 5, each dataset represents BOLD responses collected from an individual subject. In this case, the samples correspond to the timepoints of BOLD responses, and the dimensions correspond to voxels.

To create the datasets, we first randomly initialized two latent variables and two independent components. We then constructed each of the two datasets by combining both latent variables and one of the independent components. If Pyrcca works as expected then it should capture the relationship between the dataset by recovering two canonical components corresponding to the two latent variables.

This example is implemented in a Jupyter notebook included in the Pyrcca GitHub repository (http://github.com/gallantlab/pyrcca). We encourage the reader to use the notebook to explore this example interactively.

### 4.1. Pyrcca usage example analysis

Two interdependent datasets with 1000 samples were constructed by combining two latent variables and additional independent components. The first dataset had four dimensions, and the second dataset had five dimensions. Each dimension of each dataset was constructed as a weighted sum of an independent component (25%) and one of the two latent variables (75%). The first latent variable was used to construct dimensions 1 and 3 of the first dataset and dimensions 1, 3, and 5 of the second dataset. The second latent variable was used to construct dimensions 2 and 4 of both the first and the second dataset. The independent components and the latent variables were all drawn randomly from a Gaussian distribution using the numpy.random.randn() method. The code below shows how the latent variables and independent noise components were initialized and how the datasets were created.


  import numpy as np
  nSamples = 1000
  latvar1 = np.random.randn(nSamples,)
  latvar2 = np.random.randn(nSamples,)
  indep1 = np.random.randn(nSamples, 4)
  indep2 = np.random.randn(nSamples, 5)
  data1 = 0.25*indep1 + 0.75*np.vstack((latvar1,
       latvar2, latvar1, latvar2)).T
  data2 = 0.25*indep2 + 0.75*np.vstack((latvar1,
       latvar2, latvar1, latvar2, latvar1)).T


Each dataset was divided into two halves: a training set and a test set. The code below shows how the datasets were split:


  train1 = data1[:nSamples/2]
  train2 = data2[:nSamples/2]
  test1 = data1[nSamples/2:]
  test2 = data2[nSamples/2:]


Pyrcca was used to estimate a CCA mapping between the two training datasets. Kernelization and regularization were not used. The maximum possible number of canonical components (four) was found. The quality of the mapping was quantified using cross-dataset prediction with the test datasets. The code below shows how the analysis was implemented:


  import rcca
  nComponents = 4
  cca = rcca.CCA(kernelcca = False,
       reg = 0., numCC = nComponents)
  cca.train([train1, train2])
  testcorrs = cca.validate([test1, test2])


### 4.2. Pyrcca usage example results

The results of the analysis were evaluated in two ways. First, we examined the canonical correlations to determine the number of meaningful canonical components recovered by Pyrcca. Second, we quantified cross-dataset prediction performance to determine whether the mapping estimated by Pyrcca was valid for held-out data.

The first two canonical correlations were both 0.95, while the third and the fourth canonical correlations were 0.10 and 0.00, respectively. This result shows that the first two canonical components capture meaningful relationships between the datasets, while the third and the fourth canonical components do not. Cross-dataset prediction with test datasets was highly accurate. The correlations of the predicted and actual held-out data ranged from 0.90 to 0.93 for each dimension of the two datasets. This result shows that the mapping estimated by Pyrcca is valid for held-out datasets that depend on the same latent variables.

Taken together, these results show that Pyrcca recovers the structure of the relationships between the datasets defined by the two latent variables.

### 4.3. Pyrcca usage example with cross-validation

It is possible to use cross-validation to find the optimal regularization coefficient and the optimal number of components empirically. In the analysis described in Section 4.2, the regularization coefficient was set to 0. However, it may be useful to use regularization this analysis to relax the orthogonality constraint between the canonical components. Because the latent variables were randomly drawn from a Gaussian distribution, they may not be orthogonal. Thus, regularized CCA may be optimal for capturing the true structure of the similarities between the datasets. We tested four values for the regularization coefficient: 0, 10^2^, 10^4^, and 10^6^.

Additionally, in the analysis described in Section 4.2, the canonical correlations showed that the first two canonical components captured meaningful relationships between the datasets, whereas the third and the fourth component did not. We used cross-validation to test all possible numbers of canonical components: 1, 2, 3, and 4, to verify that two components is indeed optimal.

The code below shows how the analysis with cross-validation was implemented:


  ccaCV = rcca.CrossValidate(kernelcca = False,
       numCCs = [1, 2, 3, 4],
       regs = [0, 1e2, 1e4, 1e6])
  ccaCV.train([train1, train2])
  testcorrsCV = ccaCV.validate([test1, test2])


The analysis was run 1000 times, with random data generated on each iteration. The optimal regularization coefficient based on cross-validation results varied for different initializations of the data, but it was greater than zero for over 90% of the iterations. The variation of the optimal regularization coefficient was expected because the level of orthogonality between the latent variables varies for each instantiation.

The optimal number of components was two for 97% of the iterations, based on cross-validation results. This result was consistent with the findings described in Section 4.2 and showed that Pyrcca was able to recover the relationships between the datasets predefined by the two latent variables.

The canonical correlations and test set prediction correlations were comparable to the analysis with predefined hyperparameters described in Section 4.2. Canonical correlations were 0.95 for both components. The test set prediction correlations ranged between 0.90 and 0.94 for each dimension of the datasets.

The example described here is abstract by design. It is merely intended to demonstrate how Pyrcca can be used to describe relationships between any timeseries data. In the next section, we show how Pyrcca can be applied to a concrete data analysis problem in neuroimaging.

## 5. Cross-subject comparison in fMRI using Pyrcca

CCA has many potential applications for neuroimaging data analysis. In this article, we focus on one particular neuroimaging analysis problem: cross-subject comparison in an fMRI experiment. In a typical fMRI study, data are collected from multiple participants. Thus, there is a pressing need to compare and combine data across individuals. The most common method for comparing measurements from individual brains is to resample the spatiotemporal data from individual subjects to a common anatomical template. These resampled, transformed data are then averaged to obtain a group map. This procedure increases statistical power in regions of the brain where the transformation tends to aggregate signal across individuals, but it decreases power in brain regions that are more variable across individuals. Signal variability stems from two sources: structural differences in brain anatomy and differences in BOLD (blood oxygen level dependent) signal intensity. Both anatomical and functional variability complicates results obtained by anatomical normalization.

To improve anatomical template registration, most modern fMRI studies use nonlinear registration algorithms that optimize alignment of brain curvature across subjects (Greve and Fischl, 2009; Fischl, 2012). However, these anatomical methods do not address functional variation in BOLD signal that is less directly tied to the underlying anatomy. There are several cross-subject alignment methods that instead rely on correlations between functional responses, such as hyperalignment and similarity space alignment (Haxby et al., 2011; Raizada and Connolly, 2012; Conroy et al., 2013). However, these methods usually require anatomical template registration as a precursor to analysis. They also assume a voxel-to-voxel correspondence of brain patterns across subjects. Additionally, these methods do not reveal the underlying structure of the similar brain responses, but only quantify their similarity.

Cross-subject comparison by CCA can find underlying relationships among datasets recorded from different subjects in the same experiment. Because CCA does not require datasets to have equal dimensionality, individual subject data do not need to be resampled to an anatomical template before analysis. Furthermore, the resulting canonical coordinate space can be used to obtain a clear interpretation of the underlying similarities in fMRI responses of individual subjects.

In this section, we demonstrate how to use Pyrcca software to perform CCA on neuroimaging data. We used Pyrcca to perform cross-subject comparison of fMRI data collected from three individuals while they watched natural movies (Nishimoto et al., 2011). This dataset is available publicly (Nishimoto et al., 2014). We estimated canonical components across subjects in order to identify commonalities in patterns of brain responses. To provide further evidence of the veracity of our results, we then used the recovered canonical component space to predict each individual subject's responses to novel movies based on the other subjects' responses. Finally, we examined resulting canonical weights on each subject's cortical surface and found that the canonical components revealed retinotopic organization in each subject.

The code for running the analyses described in this section is implemented in a Jupyter notebook that is included in the Pyrcca GitHub repository (http://github.com/gallantlab/pyrcca). The user should be aware, however, that this is a computationally intensive analysis that will take a very long time to run on a single desktop computer. The full analysis presented here was run on a distributed computing cluster.

### 5.1. fMRI experiment

The design and methods of the fMRI experiment were described in detail in an earlier publication from our laboratory (Nishimoto et al., 2011). In brief, fMRI responses were recorded from three subjects who watched natural movies in a 4 Tesla Varian MRI scanner at UC Berkeley. Functional BOLD responses were collected at 1 Hz. The scanning volume covered the posterior-ventral quarter of the head with a 64 × 64 × 18 matrix. The analysis included only cortical voxels for each subject. The cortical voxels were identified by manually aligning functional and anatomical volumes for each subject in Pycortex (Gao et al., 2015) and then selecting the functional voxels that overlapped with the anatomical cortical mask. This procedure produced 34,407 voxels for subject 1, 30,373 voxels for subject 2, and 33,356 voxels for subject 3.

The functional data were corrected for subject motion in FSL (Jenkinson and Smith, 2001; Jenkinson et al., 2002; Greve and Fischl, 2009) before alignment with the anatomical volume. Median detrending was used to remove low-frequency noise from the data. Training and test data for each subject were collected in alternating scans. The training movies were shown once. The test movies were shown ten times, and the responses were averaged to increase signal to noise ratio. The training responses spanned 7200 timepoints (7200 s), and test responses spanned 540 timepoints (540 s) after averaging. The subjects provided written informed consent. The experimental protocol was approved by the Committee for the Protection of Human Subjects at University of California, Berkeley.

### 5.2. Cross-subject comparison methods

Pyrcca was used to find a cross-subject CCA mapping among the training BOLD responses of the three experimental subjects. To reduce the computational complexity of the analysis, a linear (inner product) kernel was used. Regularization was used because of the kernelization and because the number of dataset dimensions (voxels) outnumbered the number of dataset samples (timepoints). The optimal hyperparameters for the analysis were chosen using grid search with cross-validation. The optimal regularization parameter was chosen from a logarithmically spaced range of ten values between 1 × 10^−4^ and 1 × 10^2^. The optimal number of components was chosen from a linearly spaced range of eight values between 3 and 10 components. We selected these ranges based on pilot analyses performed on an independent dataset that was not used for this publication.

To initiate the analysis, an instantiation of the class rcca.CCACrossValidate was created with the hyperparameters described above. The CCA mapping was estimated using the train() method with the training BOLD responses for all three subjects. The mapping was tested by performing cross-dataset prediction on the held-out test BOLD responses, using the validate() method. Finally, to evaluate the influence of each canonical component on the BOLD responses of each subject across the cortical surface, the explained variance for each voxel was quantified using the compute_ev() method for all three subjects. The explained variance was evaluated using the held-out test BOLD responses. The analysis code is shown below.


  import rcca
  cca = rcca.CCACrossValidate(kernelcca = True,
       regs = np.logspace(-4, 2, 10),
       numCCs = np.arange(3, 11))
  cca.train([training_data1, training_data2,
       training_data3])
  corrs = cca.validate([test_data1, test_data2,
       test_data3])
  ev = cca.compute_ev([test_data1, test_data2,
       test_data3])


### 5.3. Cross-subject comparison results

Cross-validation was used to determine the optimal hyperparameters. The optimal regularization coefficient was 0.01, and the optimal number of canonical components was 3. The results of the analysis were evaluated in three ways: by quantifying cross-subject prediction, by examining the canonical weight maps, and by examining explained variance maps for each canonical component.

#### 5.3.1. Cross-subject prediction

The results of cross-subject prediction on held-out data were examined by plotting the voxelwise correlations of the actual and predicted BOLD responses on the cortical maps of the subjects. The correlations for each subject were also plotted as a histogram. To evaluate whether the prediction accuracy was significant, the correlations were subjected to an asymptotic significance test.

**Figure 2:**
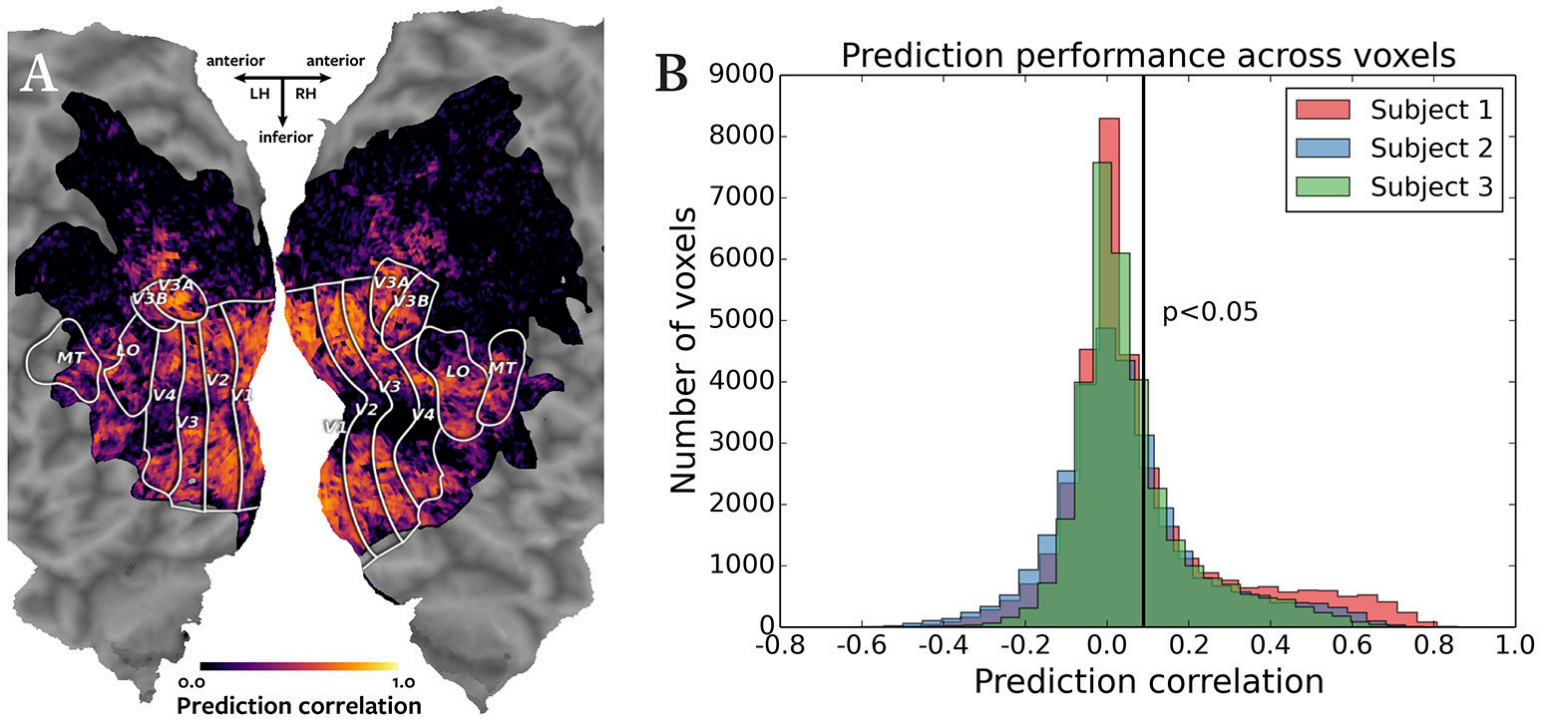
**Accuracy of cross-subject prediction with Pyrcca. (A)** Cross-subject prediction performance for subject 1 plotted on a flattened cortical map. The cortical map was created by digitally inflating the cortical surface of each hemisphere of the brain of subject 1, and then making relaxation cuts to create a flat map. Only the occipital lobe is shown here. Known regions of interest were identified in a separate retinotopic mapping experiment and are outlined in white. Each location in the cortical map represents a single voxel. The color of each voxel corresponds to the correlation between the held-out BOLD responses and responses predicted from the corresponding BOLD responses of subjects 2 and 3, and the estimated canonical components. Correlations for voxels in which prediction accuracy fell below the significance threshold (*p* < 0.05, corrected for multiple comparisons) are set to 0. The subject's responses are well predicted for voxels throughout the visual cortex. **(B)** Cross-subject prediction performance for all subjects plotted as an overlayed histogram. The correlations for subject 1 are plotted in red, the correlations for subject 2 are plotted in blue, and the correlations for subject 3 are plotted in green. The black vertical line indicates the threshold of statistical significance (*p* < 0.05, corrected for multiple comparisons). Cross-subject prediction accuracy is consistent across subjects. This figure demonstrates that Pyrcca can be used to accurately predict BOLD responses to novel visual stimuli based on cross-subject similarity.

Figure 2 shows the results of the cross-subject prediction. Panel A shows the cortical map for subject 1, with the color of each voxel representing the correlation of the predicted and actual responses for that voxel. The predicted responses in the visual cortex voxels were highly accurate, as expected in a natural movie experiment. Panel B shows an overlayed histogram of the prediction correlation values for all three subjects, with correlation values for each subject plotted in a different color. The prediction performance is consistent across subjects. Based on the asymptotic significance test of the prediction correlations, 11,134 voxels were predicted significantly for subject 1, 9158 voxels for subject 2, and 9360 voxels for subject 3 (*p* < 0.05, corrected for multiple comparisons using False Discovery Rate). Significant accuracy of cross-subject predictions demonstrates that Pyrcca can be used to predict BOLD responses to novel visual stimuli based on cross-subject similarity and without an explicit model.

#### 5.3.2. Canonical weight maps

The canonical components estimated by Pyrcca were examined by plotting the voxelwise canonical weights on the subjects' cortical maps. Three canonical components were estimated in the analysis, making it possible to use a single cortical map to visualize all canonical components at once. One color channel (red, green, or blue) was assigned to each canonical component and the canonical weights for all three canonical components for each voxel were plotted using an RGB colormap.

**Figure 3:**
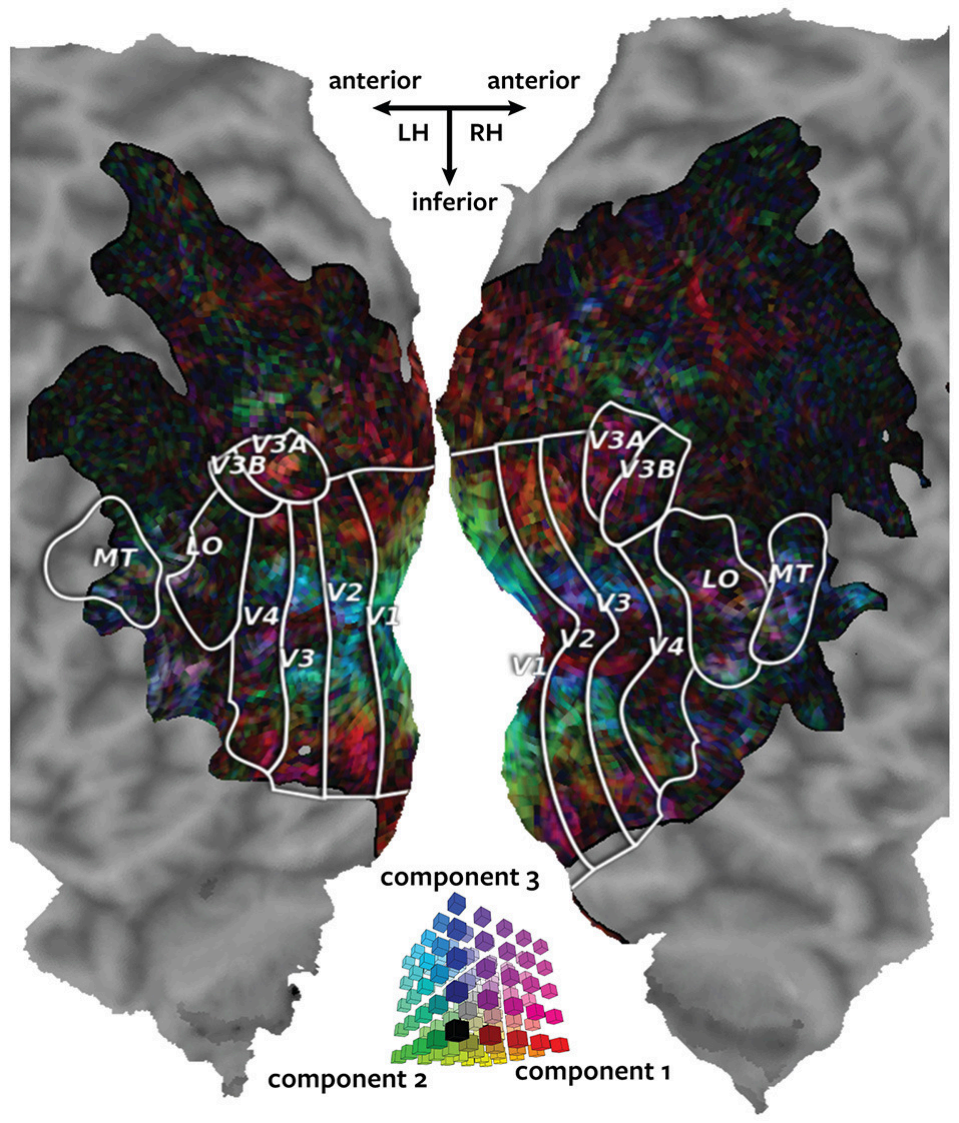
**Cortical map of voxelwise canonical weights**. The canonical weights for all three canonical components estimated by Pyrcca are shown on a flattened cortical map for subject 1. Each of the canonical components is assigned to one color channel. The first canonical component is represented by the red channel, the second canonical component is represented by green, and the third canonical component is represented by blue. Thus, the color of each voxel reflects its canonical weights for all three canonical components, as shown in the three-dimensional RGB colormap at the center of the figure. Canonical weights have been rescaled to span the range from zero to one, and the absolute value of the weights has been taken. This map shows how the BOLD responses of each voxel are described by the three canonical components. The recovered map reveals retinotopic organization of the visual cortex.

Figure 3 shows the canonical weights for all three canonical components estimated by Pyrcca plotted on the cortical map for subject 1. The red channel represents the voxel's canonical weight for the first canonical component, the green channel represents the voxel's canonical weight for the second canonical component, and the blue channel represents the voxel's canonical weight for the third canonical component. The ranges of the canonical weights were balanced by rescaling each set of the canonical weights to span the range from zero to one. The absolute value of the canonical weights was taken to adequately visualize the contribution of the negative and positive weights. The resulting color of each voxel shows how much its response is described by each of the three canonical components in relation to one another.

The three canonical components estimated by Pyrcca capture distinct retinotopic patterns in the BOLD responses. Red voxels are primarily described by the first component. These tend to be located in retinotopic areas that represent the periphery of the visual field. Green voxels are primarily described by the second component. These are located in V1, the first stage of visual processing in the cerebral cortex. Blue voxels are primarily described by the third component. These tend to be located in the foveal retinotopic areas and in area MT+, a motion-selective cortical region. Purple voxels (red and blue combined) are described by both the first and the third component. These tend to be located in MT+ and the intraparietal sulcus, areas that process visual motion and that regulate spatial attention.

#### 5.3.3. Explained variance maps

Each canonical component was visualized individually by plotting the canonical weights on the subjects' cortical maps, together with the variance of the held-out responses for each voxel that was explained by that canonical component.

**Figure 4:**
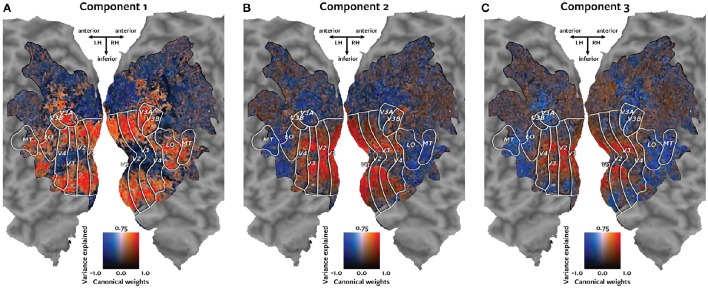
**Cortical maps of canonical weights and variance explained by each canonical component**. Each panel shows both the canonical weights for one of the estimated canonical components and the variance of the held-out BOLD responses that was explained by that canonical component. Each voxel is colored according to a two-dimensional colormap shown in the center of each panel. The hue represents the canonical weight of each voxel. Blue indicates negative canonical weights, white indicates zero weights, and red indicates positive canonical weights. The canonical weights have been rescaled to span the range from −1 to 1. The brightness reflects the variance of each voxel's held-out BOLD responses that is explained by that canonical component. The variance ranges from 0 to 75%. **(A)** The first component best explains responses of the voxels that represent the visual periphery. **(B)** The second component best explains a contrast between responses of voxels located in V1 and those located in MT+ and intraparietal sulcus. **(C)** The third component explains a contrast between voxels that represent the visual fovea and those located in MT+ and intraparietal sulcus.

Each panel in Figure 4 shows one of the canonical components visualized on the cortical map of subject 1. Each voxel is colored according to a two-dimensional colormap. The hue of the voxel represents its canonical weight for one canonical component. The hue ranges from blue for negative weights, to white at zero, to red for positive weights (note that the contrast between the negative and positive weights is meaningful, but the sign is arbitrary). The brightness of each voxel represents the variance of the held-out BOLD responses of that voxel that could be explained by that canonical component. The variance ranges from 0 to 75%. The resulting maps demonstrate how well each voxel's response can be described by each of the canonical components.

The first component, plotted in panel A, best explains responses of the voxels that represent the visual periphery. The second component, plotted in panel B, best explains a contrast between voxels located in V1 and voxels located in MT+ and intraparietal sulcus. The third component, plotted in panel C, explains a contrast between voxels that represent the visual fovea and those located in MT+ and intraparietal sulcus.

#### 5.3.4. Summary of results

Taken together, these findings reveal the similarity of BOLD responses across individual subjects. The prediction correlation map in Figure 3 demonstrates that novel BOLD responses to natural movies can be predicted based on cross-subject similarity. The canonical weight map in Figure 3 describes the variation of the BOLD responses to natural movies in terms of the estimated canonical components. The maps in Figure 4 describe the contribution of each canonical component to the variation in BOLD responses. These maps reveal retinotopic variation in the responses. Pyrcca allows us to uncover interpretable dimensions of shared BOLD responses to a complex visual stimulus in a data-driven way, without imposing an explicit model.

## 6. Conclusion

In this article, we introduce Pyrcca, a Python module for performing regularized kernel canonical correlation analysis, with a cross-validation method for hyperparameter selection. Pyrcca can be used to quantify similarity across datasets and to predict novel data via cross-dataset mapping. We demonstrate Pyrcca on an artificial example, where we use it to estimate linear relationships between two datasets. In a second example, we show how Pyrcca can be used to find shared dimensions of individual subject fMRI responses to a natural movie experiment. These dimensions are interpretable and can be used to predict novel subject responses to a held-out stimulus.

Cross-subject comparison demonstrates only one application of Pyrcca to neuroimaging data analysis. There are many neuroimaging questions that can be addressed by using Pyrcca to find relationships between interdependent neuroimaging datasets. For example, BOLD responses for one subject could be compared between different experiments to find similarities in the effects of different tasks and stimuli on brain responses. Responses measured using various imaging methods, such as fMRI, electroencephalography (EEG), and electrocorticography (ECoG), could be compared using Pyrcca for the same individual and the same task.

Although we focus on neuroimaging data analysis applications, Pyrcca can be used to analyze timeseries data in any scientific domain. We hope that researchers will find Pyrcca suitable for a variety of analysis objectives.

## Data sharing

The Pyrcca software presented in this article is available on a shared GitHub repository: http://github.com/gallantlab/pyrcca. The examples described in the article can be found in a Jupyter notebook in the GitHub repository. The fMRI data analyzed in this article are available on a shared public repository: http://crcns.org (Nishimoto et al., 2014).

## Author contributions

NB wrote the Pyrcca software, designed and conducted the analyses, and created the figures. JG supervised the research. NB and JG wrote the manuscript.

## Funding

This work was supported by grants from the National Eye Institute (EY019684) and from the Center for Science of Information (CSoI), an NSF Science and Technology Center, under grant agreement CCF-0939370. NB was additionally supported by the NSF Graduate Research Fellowship Program (1000089083).

### Conflict of interest statement

The authors declare that the research was conducted in the absence of any commercial or financial relationships that could be construed as a potential conflict of interest.
